# ER-SURF: Riding the Endoplasmic Reticulum Surface to Mitochondria

**DOI:** 10.3390/ijms22179655

**Published:** 2021-09-06

**Authors:** Christian Koch, Maya Schuldiner, Johannes M. Herrmann

**Affiliations:** 1 Cell Biology, University of Kaiserslautern, 67663 Kaiserslautern, Germany; c_koch@rhrk.uni-kl.de; 2 Department of Molecular Genetics, Weizmann Institute of Science, Rehovot 7610001, Israel; maya.schuldiner@weizmann.ac.il

**Keywords:** chaperones, contact sites, endoplasmic reticulum, ER-SURF, membrane extraction, mitochondria, protein targeting

## Abstract

Most mitochondrial proteins are synthesized in the cytosol and targeted to the mitochondrial surface in a post-translational manner. The surface of the endoplasmic reticulum (ER) plays an active role in this targeting reaction. ER-associated chaperones interact with certain mitochondrial membrane protein precursors and transfer them onto receptor proteins of the mitochondrial surface in a process termed ER-SURF. ATP-driven proteins in the membranes of mitochondria (Msp1, ATAD1) and the ER (Spf1, P5A-ATPase) serve as extractors for the removal of mislocalized proteins. If the re-routing to mitochondria fails, precursors can be degraded by ER or mitochondria-associated degradation (ERAD or MAD respectively) in a proteasome-mediated reaction. This review summarizes the current knowledge about the cooperation of the ER and mitochondria in the targeting and quality control of mitochondrial precursor proteins.

## 1. Introduction

It is the hallmark of eukaryotic cells that intracellular membranes define multiple functionally different compartments. As a consequence, many, in some cell types even most, proteins that are initially synthesized in the cytosol have to leave the cytosol to reach another cellular compartment [1,2,3]. Thus, eukaryotic cells face the challenge to specifically direct thousands of proteins to their respective position and, equally important, to remove those proteins that get stranded at foreign and inappropriate locations. While localization signals, targeting factors, receptors, and translocases for many of the residents of the different organelles were identified, we are only starting to unravel how chaperones, proteases, retro-translocases, extractors, and other “correction factors” marshal and proofread the sorting of proteins to ensure well-defined proteomes and, hence, functional cellular compartments. As if this disorder was not complicated enough, recent studies suggest that the surfaces of different organelles actively cooperate in the sorting, the targeting and the clean-up of translocation intermediates on the passage to their final residence. In particular, the relevance of the endoplasmic reticulum (ER) as the professional cellular sorting station is not restricted to proteins that enter the secretory pathway, but also supports nascent proteins destined to mitochondria, peroxisomes, lipid droplets, and chloroplasts [4,5,6,7,8,9,10]. In this review, we will provide an overview of the role of the ER in targeting and degradation of mitochondrial precursor proteins.

## 2. Targeting and Translocation of Mitochondrial Proteins

Mitochondria contain a small genome coding for a handful of proteins, most of which represent hydrophobic core subunits of the respiratory chain; these proteins are presumably difficult to import from the cytosol, and their expression in mitochondria allows organelle-controlled synthesis [11,12,13]. The vast majority of mitochondrial proteins, many hundreds to thousands, are encoded in the nucleus, synthesized in the cytosol and subsequently targeted and imported into the organelle (for review see [14]). 

**Figure 1:**
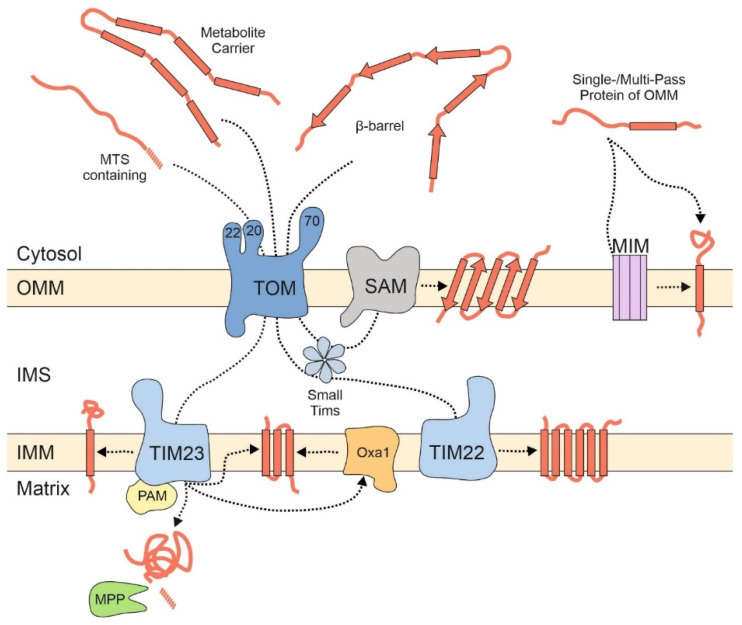
Different groups of mitochondrial proteins embark on different import pathways. Proteins of the matrix and many inner membrane proteins are synthesized as precursor proteins with N-terminal matrix targeting signals (MTSs) and imported via the TOM and TIM23 complexes. The PAM complex serves as motor for their translocation reaction. The mitochondrial processing peptidase (MPP) removes the MTS of most of these proteins. Metabolite carriers lack presequences and are integrated into the inner membrane by the TIM22 complex. The SAM complex integrates β-barrel proteins into the outer membrane. Many outer membrane proteins with helical transmembrane domains bypass the TOM complex but can be dependent on the MIM complex. IMM, inner mitochondrial membrane; IMS, intermembrane space; OMM, outer mitochondrial membrane.

Most of these proteins are synthesized with N-terminal presequences that serve as a matrix targeting signal (MTS) [15,16]. MTSs are amphipathic helices with one positively charged and one hydrophobic surface [17]. They are recognized at the mitochondrial outer membrane by three receptor proteins, Tom20, Tom22, and Tom70 that are part of the TOM (translocase of the outer membrane) complex. However, the association of Tom70 with the TOM complex is presumably dynamic and short-lived, potentially to gather the substrates for the import pore [18,19,20]. The three receptors, which differ in their exact substrate preference, pass precursor proteins in a cooperative manner to the protein-conducting channel formed by the β-barrel protein Tom40. Tom40 serves as a general entry gate for all proteins destined to the matrix, the inner membrane, the intermembrane space (IMS), and for many outer membrane proteins. After translocation through Tom40, precursor proteins are sorted to their respective submitochondrial localizations (Figure 1): Matrix and many inner membrane proteins are directed to the TIM23 (translocase of the inner membrane) complex which threads presequence-containing proteins through the inner membrane. The transfer into the matrix is promoted by the PAM (presequence translocase-associated motor) machinery by hydrolysis of matrix ATP [21] and is followed by the proteolytic removal of the presequence mediated by the mitochondrial processing peptidase, MPP.

Carrier proteins (also referred to as the SLC25A or metabolite carrier family) are highly abundant proteins of the mitochondrial inner membrane that mediate the exchange of ATP and metabolites between mitochondria and the cytosol. They usually contain six transmembrane domains and do not contain an MTS but use internal targeting signals that are scattered across their sequence [22]. These types of proteins are first recognized on the mitochondrial surface by Tom70, which also tightly cooperates with the chaperone system of the cytosol [23,24]. Soluble chaperone complexes in the IMS, formed by small TIM proteins, and the TIM22 complex then integrate carrier proteins into the inner membrane [25,26].

Many proteins of the IMS lack presequences and their import relies on cysteine motives [27]. The oxidoreductase Mia40 (CHCHD4 in humans) and the sulfhydryl oxidase Erv1 (ALR in human) drive the import reaction of these proteins [28,29].

In the outer membrane, β-barrel proteins form large pores that allow the facilitated diffusion of molecules up to a mass of several kDa. Precursors of β-barrel proteins are first recognized at the TOM complex by their β-hairpin element. These β-barrel proteins are imported through the TOM pore and inserted into the outer membrane by the SAM (sorting and assembly) machinery in a reaction that is conserved between bacteria and mitochondria [30,31].

Tail-anchored (TA) outer membrane proteins, that harbor only a single transmembrane domain (TMD) in their very C terminus, bypass the pore in the TOM complex. The mechanisms and factors that promote their insertion into the outer membrane are still poorly defined [32].

The individual steps of these import reactions were elucidated by use of very powerful in vitro import assays for which radiolabeled precursor proteins were mixed with isolated mitochondria. Whereas this approach is very well suited to study protein translocation across the mitochondrial membranes, it does not reveal the initial targeting process that occurs before precursors reach the mitochondrial surface receptors.

## 3. Productive Targeting via the ER Surface: ER-SURF

In the context of protein biogenesis, protein targeting (the passage of nascent precursors from the ribosome to the mitochondrial surface) has to be distinguished from protein translocation (which refers to the threading of precursors through mitochondrial protein translocases) [33]. Whereas mitochondrial protein translocation was studied extensively over the last two decades, mitochondrial protein targeting is by far less understood. Many recent studies documented the general importance of the cytosolic chaperone network and the ubiquitin-proteasome-system (UPS). However, we know very little about which chaperones and which ubiquitin ligases interact with which types of precursors and how their directional movement to and subsequent release from the mitochondrial membrane is mediated. Furthermore, it is unclear whether these quality control systems usher every single precursor protein along its way to the mitochondrial surface or whether they only deal with the fraction of stranded or structurally compromised precursor proteins. Thus, the early reactions of mitochondrial protein biogenesis still await to be discovered. Several recent reviews discussed these issues in depth [33,34,35]. In this article, we therefore specifically focus on the relevance of the ER surface for mitochondrial protein biogenesis.

A large fraction of all ribosomes is bound to the surface of the ER. Ribosome-bound nascent chains that expose highly hydrophobic signal sequences or transmembrane domains are recognized by the signal recognition particle (SRP) and recruited to the ER surface. Many mitochondrial proteins contain transmembrane domains, and therefore, it is not surprising that mitochondrial membrane proteins were also identified among the SRP clients [36]. For example, transmembrane domains of the inner membrane proteins Oxa1 and Psd1 were found to be recognized by the SRP. Both proteins are synthesized with presequences and use the TIM23-mediated import pathway [37,38]. However, presumably due to the lower hydrophobicity of transmembrane domains in mitochondrial membrane proteins [39,40], the SRP is able to discriminate between secretory proteins and most mitochondrial proteins. A recent study even proposed that the major mission of the SRP system is the reliable distinction of these two large groups of cellular proteins [41]. Cotranslational binders of the nascent chain, such as Ssb1/2 chaperones and the nascent chain-associated complex (NAC), fine-tune the SRP-mediated discrimination process [42,43], which might contribute to their observed relevance for mitochondrial protein biogenesis [44,45,46,47,48]. Interestingly, the factors of the guided entry of tail-anchored proteins (GET) pathway [49] also play a role in mitochondrial protein targeting. Get3, a cytosolic chaperone and targeting factor, was found to directly interact with some mitochondrial precursors, in addition to its ER-destined clients [50]. Moreover, if mitochondrial precursor proteins accumulate in the cytosol, they can be “rescued” by the GET pathway, which directs them onto the ER surface from where they finally reach the mitochondrial import machinery; this GET-mediated detour might be particularly relevant for carrier proteins and prevents their incorporation into non-productive protein aggregates [51].

**Figure 2:**
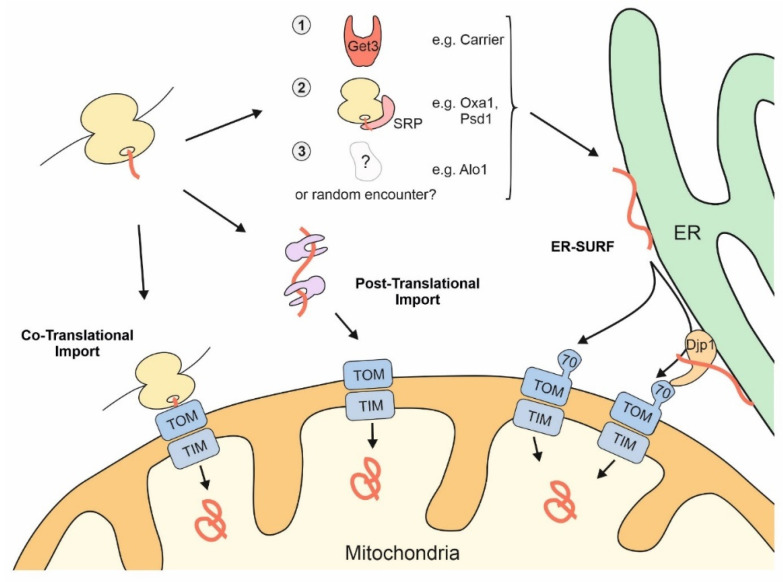
The surface of the ER facilitates mitochondrial targeting of proteins that use the ER-SURF pathway. Precursor proteins can reach mitochondria during, or after, their synthesis on cytosolic ribosomes. Some mitochondrial precursors are directed to the ER surface. For example, metabolite carriers were observed to be bound by Get3, a chaperone that facilitates ER-targeting of TA proteins. Some mitochondrial membrane proteins, such as Oxa1 and Psd1, are recognized by the SRP. For other mitochondrial proteins, such as Alo1, targeting factors were not identified so far. Djp1 is an ER-associated protein that facilitates the transfer of ER-bound proteins to mitochondria.

Thus, mitochondrial proteins might initially find themselves stranded at the ER due to “mis-localization” if the SRP and cellular quality control components are not effective enough in recognizing them. However, alternatively and not mutually exclusive, some mitochondrial proteins might deliberately associate with the ER surface or be synthesized by ER-bound ribosomes, in line with a considerable number of nascent mitochondrial proteins that were observed on the ER by ribosome profiling experiments [52,53]. Regardless of whether ER targeting of nascent mitochondrial proteins is an active, intentional mechanism or a cellular mistake, cells have evolved a pathway to help such precursors make it to their final destination. This stopover-mediated targeting route to mitochondria via the ER surface was termed the ER-SURF pathway (Figure 2) and the ER-bound J protein Djp1 was identified as a component that increased the efficiency of this import route [5,54]. The ER-SURF model proposes that the ER surface acts as an antenna that helps to funnel precursors to mitochondria. Consistent with this idea, in in vitro experiments the addition of ER fractions increases the import rate of proteins into isolated mitochondria [5,55], an observation that can hardly be reconciled with the idea that ER binding is synonymous with non-productive mis-localization. 

When mitochondrial import sites are limiting so that precursor proteins accumulate outside of mitochondria, a large number of precursors of mitochondrial membrane proteins were found to associate with the ER surface [56] and to induce the unfolded protein response pathway of the ER [57]. Since these precursor proteins, in particular those of the carriers, have a highly toxic potential [24,58,59], ER binding might serve as a safeguard mechanism [51]. This is because the ER surface is coated by a number of cellular chaperones. For example, Ydj1, the most abundant DnaJ-type co-chaperone of yeast cells, is tethered to the ER surface by a farnesyl anchor [60]. The relevance of Ydj1 for mitochondrial protein biogenesis is well documented [61,62,63], but it is not known at which intracellular localization Ydj1 “meets” mitochondrial precursors.

Djp1 forms a complex with Tom70 [64]. It is not clear whether the Djp1 species that partners Tom70 is bound to the ER. However, the ability of Tom70 to form mitochondria-ER contact sites is well established [20,65,66]. It still needs to be elucidated whether Djp1 hands over precursors from the ER to the mitochondrial surface via direct contacts or, alternatively, releases them to the cytosolic chaperone system.

A recent study in mammalian cells showed that Bcl2 can be transferred from the ER to the mitochondrial surface in a Tom20-mediated reaction that occurs at ER-mitochondria contact sites dubbed mitochondria-associated membranes (MAMs) [67]. This indicates that the ER-SURF pathway also exists in mammalian cells and that the close collaboration of the ER and mitochondria in protein sorting is a conserved feature of eukaryotic cells.

## 4. Contact Sites of the ER and Mitochondria 

A function for the close cooperation of ER and mitochondria via membrane contact sites was identified three decades ago when MAMs were first purified [68,69]. The MAM was described as a membranous fraction that was inseparable even from highly purified mitochondria and found to play a role in phospholipid biosynthesis. However, molecular tethers that connect mitochondria to the ER remained unclear until the ERMES (ER mitochondria encounter structure) complex was identified in budding yeast in 2009 [70]. This complex consists of the four structural components Mmm1, Mdm12, Mdm34, and Mdm10, which form a chain-like bridge holding the two organelles in close proximity [70,71,72,73]. ERMES is mainly involved in the transfer of phophatidylserine from the ER to mitochondria where it is converted to phosphatidylethanolamine. Deletion of any ERMES component leads to a decrease in the rate of phosphatidylethanolamine synthesis and the overall levels of cardiolipin in mitochondria and to a collapse of the mitochondrial network [74,75,76,77,78]. Moreover, ERMES promotes the formation of mitochondria-derived compartments (MDCs) [6], defines the position of intra-mitochondrial complexes such as nucleoids, the MICOS (mitochondrial contact site and cristae organizing center), and the coenzyme Q synthome [78,79,80]. 

In addition to the ERMES complex, at least two further tethering complexes connect the ER with mitochondria in yeast: One tether is formed by the ER-resident sterol transporter Lam6/Ltc1 and Tom70 [65,66], the other was proposed to form by the ER membrane complex (EMC) and Tom5 [81].

In mammalian cells, multiple tethering molecules have been suggested to act at contact sites between the ER and mitochondria [82]. For example, a single recent split proximity labeling approach proposed that 30 proteins are enriched in ER mitochondrial contact zones [83], but the individual functions of these factors still need to be elucidated; however, there is no doubt that these contacts are highly important for cellular functionality [84,85]. Thus, the ER and mitochondria form entangled intracellular networks that are connected by several specific tethering complexes. These contact sites ensure a close proximity of the ER and mitochondrial membranes and thereby support the biogenesis of (membrane) proteins and lipids.

## 5. Destructive Targeting via ERAD and MAD

**Figure 3:**
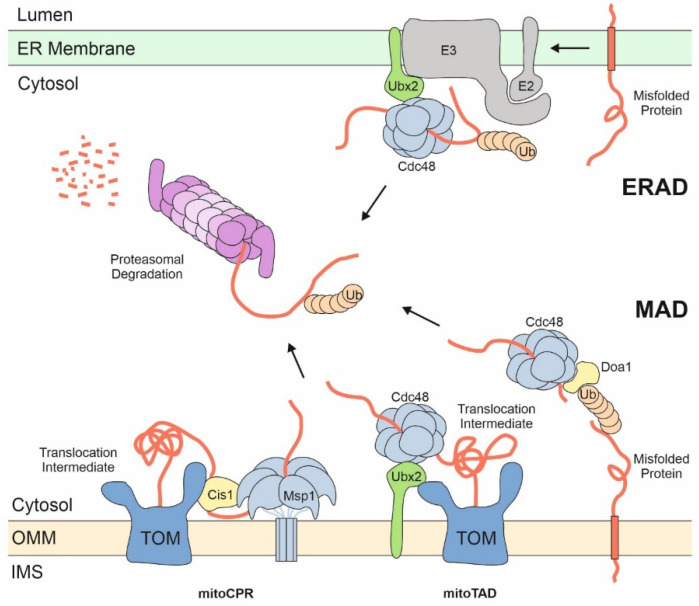
Proteasomal degradation of proteins at the ER and the mitochondrial surface. ER and mitochondrial proteins are released by the AAA proteins Cdc48/p97 (on both ER and mitochondria) or Msp1 (on mitochondria) into the cytosol to be degraded by the proteasome. Poly-ubiquitin chains serve as degradation signals and as handles on the proteins for unfolding and insertion into the proteasome. Adaptor proteins such as Ubx2 or Doa1 play crucial role in the substrate binding of AAA proteins. Stalled translocation intermediates induce the recruitment of Msp1 to the TOM complex by Cis1 in a process called mitochondrial compromised protein import response (mitoCPR). MAD of translocation intermediates is also referred to as mitochondrial protein translocation-associated degradation (mitoTAD).

Proteasomal degradation of ER and mitochondrial proteins (Figure 3) is often summarized under the umbrella terms ERAD (for ER-associated degradation) and MAD (for mitochondria-associated degradation) [86,87,88,89]. The components and underlying mechanisms of ERAD are rather well understood, and even structures of the ERAD machinery were recently published [90]. In contrast, the puzzle pieces of MAD are only in the process of being collected and therefore a comprehensive, generally accepted picture still has to emerge. 

The UPS plays a general role in the degradation of non-functional or damaged outer membrane proteins. Mitofusins (Fzo1 in yeast) were among the first mitochondrial proteasome substrates that were identified and the proteasomal turnover of these fusion factors is crucial for mitochondrial morphogenesis [91,92,93]. Surprisingly, many of the factors found to carry out MAD were initially established as ERAD factors. Both processes share the AAA unfoldase Cdc48/p97 and the adaptor proteins Ubx2 and Doa1/Ufd3 [94,95,96,97]. For example, Ubx2, a well-established ER-embedded ERAD factor, also forms a pool on the outer membrane of mitochondria. It monitors the TOM complex and in case of accumulating translocation intermediates, Ubx2 recruits Cdc48 to prevent clogging of mitochondrial import sites. This Ubx2-mediated subcategory of MAD, which is important to cleanse stalled translocation intermediates from the TOM complex, was called mitoTAD for mitochondrial protein translocation-associated degradation [98]. Doa1/Ufd3 might play an Ubx2-equivalent role in the degradation of outer membrane proteins [94,95,96,97].

At least in mammalian cells, ubiquitination might even regulate and fine-tune the import through the TOM complex: precursors are ubiquinated by the ubiquitin ligase March5 (also called MITOL) before ubiquitin is removed by the deubiquitinase USP30. If deubiquitination does not keep pace with ubiquitination, translocation intermediates are arrested in the TOM complex, resulting in their degradation [99]. The USP30-mediated deubiquitination of translocation intermediates is also relevant to prevent mitophagy via activation of the ubiquitin ligase Parkin and the protein kinase PINK1 [100]. 

## 6. The Role of Membrane Extractors

The degradation of membrane proteins and translocation intermediates by ERAD and MAD requires their extraction and presentation to the proteasome. The cytosolic AAA protein Cdc48/p97 serves as such an extractor: this hexamer consists of two stacked ATPase rings that pull substrate proteins through the central cavity, thereby generating the force required to dislodge membrane proteins [101]. As mentioned before, different adaptor proteins, such as Ubx2 and Doa1/Ufd3, facilitate the binding of ubiquitinated substrates to Cdc48/p97. 

**Figure 4:**
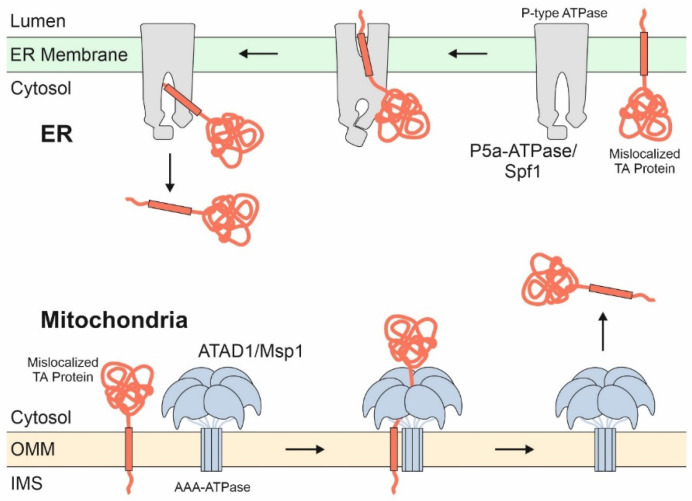
ATP-driven membrane extractors give mis-localized TA proteins a second chance to find their correct location. The ATAD1/Msp1 complex is a hexameric AAA dislocase on the outer membrane of mitochondria that recognizes non-mitochondrial TA proteins as well as translocation intermediates stalled in the TOM complex. It removes these proteins, which then either find their respective target membrane or are degraded, for example, via ERAD. An analogous extraction system exists on the ER membrane, where the P-type ATPase P5a-ATPase (also called ATP13A1 or CATP-8) recognizes and dislocates membrane proteins destined to mitochondria.

The outer membrane contains an additional AAA protein for Cdc48/p97-independent extraction. This protein complex is called Msp1 in yeast and ATAD1 in mammalian cells, shares the overall hexameric organization with Cdc48/p97 and uses a comparable mechanism for protein extraction (Figure 4). Msp1 was initially found as the dislocase for peroxisomal and ER TA proteins that were aberrantly integrated into the mitochondrial outer membrane [102,103]. After extraction these TA proteins are either sent for degradation or passed on to their cognate target membrane [8,104]. Msp1 also extracts translocation intermediates that get stuck in the import tunnel, a function for which it requires being specifically recruited to the TOM complex by the adaptor Cis1 [105,106]. Interestingly, proteins extracted from mitochondria by Msp1 can be targeted to the ER surface where they are turned over by ERAD. Once again, this emphasizes the close alliance of the ER and mitochondria during protein biogenesis.

A membrane-bound extractor is also found on the ER membrane; this protein is called Spf1 in yeast or P5A-ATPase in mammals [107,108]. It safeguards the ER by dislocation of mis-localized mitochondrial proteins, analogous to the function of Msp1/ATAD1 on mitochondria. A potential role of Spf1/P5A-ATPase in ER-SURF still has to be elucidated.

## 7. ER-Mitochondria Contact Zones as Protein Nurseries

While the ER has been known for 30 years to serve as a general sorting station for proteins of the secretory pathway, the biogenesis of proteins of mitochondria, chloroplasts, and, to some degree, peroxisomes and lipid droplets was traditionally regarded as processes that occur independently from the ER. This assumption was fueled by the observation that, in vitro, isolated proteins can be efficiently imported into mitochondria, chloroplasts, and peroxisomes in a post-translational reaction. The success of high-resolution light microscopy and cryo-electron microscopy revealed fascinating insights into the interplay of the different organelles, and many recent studies discovered the close collaborations between the intracellular networks formed by the ER and by mitochondria. Examples for such cooperations of the ER and mitochondria include the control of mitochondrial fusion and fission [109,110,111,112], the positioning of genomes within mitochondria [78], the formation of isolation membranes for autophagy [113,114,115], or the transfer of lipids [70,72]. It therefore is no surprise that ER mitochondria contact sites are also relevant for protein targeting reactions and that the surfaces of the different membranes closely collaborate for protein sorting. 

We are only beginning to appreciate the dynamic interplay of organellar surfaces in the context of protein biogenesis. These inter-organellar interactions are presumably particularly relevant in the context of the large number of dually localized proteins [116], such as Psd1 [117], DAKAP1 [118] or NADH-cytochrome b_5_ reductase [119]. 

Thus, the contact zone between mitochondria and the ER apparently serves as a nursery where nascent proteins, under surveillance of cytosolic chaperones, and the quality control factors of MAD and ERAD, find their appropriate destination membrane. It will be exciting to further explore how eukaryotic cells orchestrate these biogenesis hotspots to avoid an unproductive chaotic jumble.
